# Aberrant Expression of Circulating MicroRNA Leads to the Dysregulation of Alpha-Synuclein and Other Pathogenic Genes in Parkinson’s Disease

**DOI:** 10.3389/fcell.2021.695007

**Published:** 2021-08-23

**Authors:** Meng Cai, Songshan Chai, Tao Xiong, Jun Wei, Weibing Mao, Yasha Zhu, Xiang Li, Wei Wei, Xuan Dai, Bangkun Yang, Wen Liu, Bing Shu, Mengyang Wang, Taojunjin Lu, Yuankun Cai, Zhixin Zheng, Zhimin Mei, Yixuan Zhou, Jingyi Yang, Jingwei Zhao, Lei Shen, Joshua Wing Kei Ho, Jincao Chen, Nanxiang Xiong

**Affiliations:** ^1^Department of Neurosurgery, Zhongnan Hospital of Wuhan University, Wuhan, China; ^2^iRegene Therapeutics, Wuhan, China; ^3^Department of Neurology, Fifth Hospital in Wuhan, Wuhan, China; ^4^The Second Clinical College of Wuhan University, Wuhan, China; ^5^School of Biomedical Sciences, Li Ka Shing Faculty of Medicine, The University of Hong Kong, Pokfulam, Hong Kong

**Keywords:** PD-related genes, alpha synuclein, microRNA, Parkinson’s disease, exosomes (EX)

## Abstract

A group of circulating microRNAs (miRNAs) have been implicated in the pathogenesis of Parkinson’s disease. However, a comprehensive study of the interactions between pathogenic miRNAs and their downstream Parkinson’s disease (PD)-related target genes has not been performed. Here, we identified the miRNA expression profiles in the plasma and circulating exosomes of Parkinson’s disease patients using next-generation RNA sequencing. Kyoto Encyclopedia of Genes and Genomes (KEGG) and Gene Ontology (GO) analyses showed that the miRNA target genes were enriched in axon guidance, neurotrophin signaling, cellular senescence, and the Transforming growth factor-β (TGF-β), mitogen-activated protein kinase (MAPK), phosphatidylinositol 3-kinase (PI3K)-protein kinase B (AKT) and mechanistic target of rapamycin (mTOR) signaling pathways. Furthermore, a group of aberrantly expressed miRNAs were selected and further validated in individual patient plasma, human neural stem cells (NSCs) and a rat model of PD. More importantly, the full scope of the regulatory network between these miRNAs and their PD-related gene targets in human neural stem cells was examined, and the findings revealed a similar but still varied downstream regulatory cascade involving many known PD-associated genes. Additionally, miR-23b-3p was identified as a novel direct regulator of alpha-synuclein, which is possibly the key component in PD. Our current study, for the first time, provides a glimpse into the regulatory network of pathogenic miRNAs and their PD-related gene targets in PD. Moreover, these PD-associated miRNAs may serve as biomarkers and novel therapeutic targets for PD.

## Introduction

Parkinson’s disease (PD), the second-most common neurodegenerative disorder, is characterized by the loss of dopaminergic (DA) neurons in the substantia nigra pars compacta (SNc) and the accumulation of cytoplasmic inclusions called Lewy bodies (LBs) and Lewy neurites in surviving neurons, the pathological hallmarks of PD (Schapira, 1997). Currently, PD diagnosis mainly depends on neuroimaging and clinical manifestations using the United Parkinson’s Disease Rating Scale (UPDRS) and modified Hoehne Yahr stage (Massano and Bhatia, 2012). These dual criteria are subjective and can be applied only when motor features appear. Histopathology has become the “gold standard” for diagnosing PD, but the invasiveness of the procedure and the potential sampling error associated with random brain biopsy limit its effectiveness (Shtilbans and Henchcliffe, 2012; Reschke and Henshall, 2015). These limitations highlight the need for new non-invasive diagnostic and prognostic biomarkers of PD. In addition, the current drug therapy for PD has negligibly improved since the 1950s (Djamshidian and Poewe, 2016). Drug development strategies based on the treatment or control of motor symptoms have proven to be “symptomatic treatment” not “disease modifying” therapies (Harikrishna Reddy et al., 2014). Moreover, current DA precursors and DA agonist-based therapies have been shown to cause serious adverse effects that has hampered clinical application (LeWitt, 2015). Effort has been devoted to developing new drugs against novel therapeutic targets, such as alpha-synuclein (SNCA) and leucine-rich repeat kinase 2 (LRRK2) (Dehay et al., 2015; Chen J. et al., 2018). However, the effects of these new drugs in clinical trials yielded unsatisfactory outcomes, highlighting the urgent demand for a better understanding of the global pathogenic regulatory network in PD (Sardi and Simuni, 2019).

MicroRNAs (miRNAs) are small (approximately 22 nucleotides long) non-coding RNAs that directly regulate the posttranscriptional expression of target mRNAs (Bartel, 2004). Recently, circulating miRNAs in plasma, serum, or serum exosomes have emerged as potential biomarkers for PD diagnosis (Khoo et al., 2012; Cardo et al., 2013; Yao et al., 2018). However, most studies are observational and lack validation in PD models. In particular, the specific roles of these circulating miRNAs in PD pathogenesis and their relationships with PD-related pathogenic genes remain to be elucidated. miRNAs have been shown to regulate PD-related genes, such as SNCA, by direct or indirect effects in PD pathogenesis (Santosh et al., 2009). SNCA protein is the major component in LBs and Lewy neurites (Spillantini et al., 1998). Emerging evidence suggests that SNCA gene and protein aggregation are strongly associated with PD (Singleton et al., 2003; Olanow and Brundin, 2013), and increased SNCA expression plays a critical role in the pathophysiological process of PD. As a well-established causative gene in PD, the interactions between SNCA and other PD-related genes, such as LRRK2, Parkin RBRE3 ubiquitin protein ligase (PRKN), Parkinsonism-associated deglycase (PARK7), PTEN-induced putative kinase 1 (PINK1), and ATPase 13A2 (ATP13A2), have been extensively studied (Bekris et al., 2010; Nuytemans et al., 2010; Klein and Westenberger, 2012; Heman-Ackah et al., 2013). The literature indicates that these PD-related genes can influence the expression or biological function of SNCA to exert their pathological roles (Lin et al., 2009; Zhu et al., 2016). However, the regulatory network involving disease-related miRNAs and associated genes in PD pathogenesis remains vague.

In the current study, we aimed to characterize the expression profile of miRNAs in the plasma and exosomes of PD patients and further examine the relationship between these plasma miRNAs and PD-related genes. The identified regulatory networks, including miRNAs, SNCA and other PD-related genes, provide new insight into comprehensive PD pathogenesis. These data highlight the potential clinical applications of circulating miRNAs in molecular diagnostics and drug development in PD.

## Materials and Methods

### Patient Sample Collection and Preparation

Peripheral blood samples of PD patients and healthy volunteers were collected from the Union Hospital affiliated with Huazhong University of Science and Technology. The Institutional Ethics Committee approved the blood collection, and informed consent was obtained from the subjects. The diagnosis of PD was based on the established criteria of the Hoehn-Yahr (H-Y) staging system. Approximately 6 ml of peripheral blood samples were collected from 22 patients with PD and nine healthy volunteers. Due to the limitation of sample amount, the mixed RNA samples from multiple donors were used for RNA sequencing. In addition, seven healthy volunteer (*N* = 7) and five PD patient (*N* = 5) samples were used for subsequent quantitative PCR analysis.

### Exosome Extraction and Characterization

The exoEasy Maxi kit (Qiagen Cat. No. 76064, United States) was used to extract plasma exosomes according to the manufacturer’s protocol. The extracted exosomes were characterized by nanoparticle tracking analysis (NTA) using NanoSight NS300 and biological transmission electron microscopy (TEM) to measure the particle size distribution and morphology of exosomes. Western blotting was performed to determine specific exosome surface markers, including cluster of differentiation 9 (CD9, 1:1000; ab92726; Abcam), tumor susceptibility 101 (TSG101, 1:1000; ab83; Abcam) and heat shock cognate protein 70 (HSC70, 1:1000; ab1427; Abcam).

### Total Small RNA Extraction From Exosome and Peripheral Blood Samples

Total small RNA extraction was performed using the miRNeasy serum/plasma kit (Qiagen Cat. No. 217184, United States) and BIOG cfRNA Easy kit (Bio-generating Biotechnology, BIOG, Cat. No. 51027-MF, China) following the manufacturer’s recommendations. After total small RNA was extracted, the RNA concentration and purity were measured by a NanoDrop spectrophotometer (Thermo Fisher, United States) and LabChip GX Touch HT nucleic acid analyzer (PerkinElmer, United States).

### Library Preparation and HiSeq Sequencing

Sequencing libraries were generated by Whbioacme Co. Ltd. (Wuhan, China) using NEBNext Multiplex Small RNA Library Prep Set for Illumina^®^ (NEB, United States) following the manufacturer’s recommendations, and the index codes were added to attribute sequences to each sample. Briefly, libraries were prepared by ligating different adaptors to the total RNA followed by reverse transcription, PCR amplification and size selection using 6% polyacrylamide gels. Library quality was assessed on an Agilent Bioanalyzer 2100 system. Sequencing was performed on an Illumina NovaSeq 6000.

### Sequencing Data Analysis

Raw data (raw reads) in fastq format were first processed through in-house Perl scripts. Clean data (clean reads) were obtained by removing reads containing adapters, reads containing poly-*N* and low-quality reads. At the same time, the Q20, Q30 and GC contents of the clean data were calculated. All downstream analyses were based on clean data with high quality.

The redundant clean reads were eliminated using miRDeep2 software, and the collapsed reads were compared with the reference human genome. The index of the reference genome was built using Bowtie 1. Moreover, the collapsed reads were compared with the Rfam database using cmscan to identify small RNA species. The known miRNA sequences of species were obtained in miRBase. Differential expression analysis of two conditions/groups (three biological replicates per condition) was performed using DESeq2. A corrected P-value of 0.05 and log2(fold change) of 1 were set as the thresholds for significantly differential expression. miRNA target prediction was performed by microRNA.org^1^, PicTar^2^, and TargetScan^3^. The target genes were functionally annotated and enriched according to the predicted results using GO enrichment analysis and KEGG pathway analysis^4^.

### PD Rat Model

Eight-week-old Wistar female rats with body weights ranging from 200 to 250 g (Hubei Provincial Center for Disease Control and Prevention, China) were housed in pairs in appropriate cages under standard controlled conditions. For the surgical procedures, the rats were placed on a stereotaxic apparatus and anesthetized intraperitoneally (i.p.) with ketamine (60 mg/kg) plus medetomidine (0.4 mg/kg) and unilaterally injected with either 3 μl of vehicle (0.05% ascorbate saline; sham group, *n* = 6) or 24 μg (3 μl) 6-Hydroxydopamine hydrochloride (Sigma, H4381, 6-OHDA group, *n* = 8) in 0.05% ascorbate saline directly into the right side of the medial forebrain bundle (MFB) at the following coordinates relative to the bregma: AP = −4.4 mm, ML = −1.0 or –1.2 mm, DV = −7.8 at a rate of 0.25 μl/min using a Hamilton syringe with a 30-gauge needle (Hamilton, Bonaduz, Switzerland). The needle was left in place for 5 min before withdrawal from the brain to avoid backflow.

Lesions in the rat brain of the striatum and SNc were identified by immunohistochemistry using an antibody against tyrosine hydroxylase (TH; ab112; Abcam) 4 weeks after injection. Behavioral impairment was assessed by the cylinder test, stepping test and bilateral tactile stimulation test in PD model rats as detailed below.

### Cylinder Test

Spontaneous movement of 6-OHDA injected rat was measured by cylinder test as previously described (Kim et al., 2018). The PD model rats were placed into a transparent Perspex cylinder, and the number of wall contacts made with both left and right forelegs were recorded until the total contact reached a frequency of 20. The rats were tested two times a day for 3 days to avoid deviation during data collection. The number of wall contacts made with the ipsilateral paw was compared to that of contacts made with the contralateral paw.

### Stepping Test

The stepping test was performed according to a previous description (Olsson et al., 1995). Briefly, a rat was held by the experimenter at a 45-degree angle with only the forelimb touching the table and moved slowly by the experimenter. The number of table contacts was counted for both paws at a 1-m moving distance.

### Bilateral Tactile Stimulation Test

The method was adopted from a previous study with minor changes (Tillerson et al., 2006). The responsiveness of the PD model rats to tactile stimulation was tested as the ability to contact or remove adhesive stickers (1.2 cm in diameter) on both paws. The time duration of sticker removal was recorded for ipsilateral and contralateral paws.

### NSCs Induction, 6-OHDA Treatment and Synthetic Nucleic Acid Transfection

All procedures were approved by the ethics committee of the Union Hospital, Tongji Medical College, Huazhong University of Science and Technology, China. Cord blood was collected after receiving informed consent at Union Hospital. The CD34+ progenitor cells were isolated and expanded from cord blood, and then reprogrammed to induced pluripotent stem cells (iPSCs) using a commercially available kit (Cat #05925, Stemcell Technologies). NSCs induction was performed by iRegene Therapeutics, Wuhan, China in house according to a previously published method with modifications (Shi et al., 2012). Briefly, the induced pluripotent stem cells (iPSCs) are resuspended in STEMdiff Neural Induction Medium (Stemcell Technologies) supplemented with 10-μM Y-27632 and seeded onto Matrigel (BD Bioscience) pre-coated culture plates at a density of 10,000–25,000 cells/cm2. After seeding, the medium is replaced daily with a fresh STEMdiff Neural Induction Medium without Y-27632. ON days 6–9, the cell cultures will be confluent and ready for passage. After the first passage, the cells should be passaged once they reach ∼80% confluency and adjusted to a cell density of about 10,000–25,000 cells/cm^2^. Y-27632 should be added to the medium at day 1 of each passage to ensure the cell attachment and then removed from the medium at day 2. After the third passage, the cells should be cultured with STEMdiff Neural Progenitor Medium (Stemcell Technologies) to maintain NSC growth.

To mimic neuronal damage *in vitro*, NSCs were treated with 6-OHDA at 30, 50, and 75 μM for 24 h and subsequently collected for microRNA expression analysis.

Chemically synthesized double-stranded microRNA mimics and single-stranded inhibitors against miR-23b-3p (mimic-23b-3p and inhibitor-23b-3p), miR-30b-5p (mimic-30b-5p and inhibitor-30b-3p), and miR-195-3p (mimic-195-3p and inhibitor-195-3p) were purchased from TsingKe Biological Technology, China. NSCs were seeded in a 6-well plate at a density of 3.5 × 10^6^/well and transfected with 100 pmol synthesized microRNA mimic or inhibitor per well using Lipofectamine^TM^ stem transfection reagent following a standard protocol (STEM00001, Thermo Fisher Scientific, United States). The cell samples from three independent experiments (*N* = 3) were collected 24 h after transfection for RNA extraction and the subsequent quantitative PCR.

### Quantitative PCR

cDNA synthesis was performed using a Mir-X^TM^ miRNA First-Strand synthesis kit (TAKARA, Cat. No. 638313) according to the manufacturer’s recommendations. Total RNA (100–200 ng) was used for reverse transcription to synthesize first strand cDNA.

Quantitative PCR amplification was performed with TB Green^®^ Premix Ex Taq^TM^ II (TAKARA, Cat. No. RR820A). The PCR amplification procedure was as follows: 120 s at 95°C and 40 cycles of (20 s at 95°C, 15 s at 60°C). Sample cDNA (2–5 μL) was used as a template for PCR amplification using the miRNA-specific upstream primer, and miR-22-3p and miR-185-5p were employed as internal controls. The reaction was performed by Quantagene Q325 quantitative fluorescence PCR, and the 2^−△△*CT*^ method was used for data analysis. The miRNA-specific primer and internal control primer sequences used are shown in Table 1.

**TABLE 1:** Quantitative PCR Primer sequence for miRNA.

Gene name	Upstream primer sequence(5′-3′)
miR-23b-3p	ATCACATTGCCAGGGATTACCAC
miR-30b-5p	TGTAAACATCCTACACTCAGCT
miR-195-5p	TAGCAGCACAGAAATATTGGC
miR-195-3p	CCAATATTGGCTGTGCTGCTCC
miR-342-3p	TCTCACACAGAAATCGCACCC
miR-22-3p	AAGCTGCCAGTTGAAGAACTGT
miR-185-5p	TGGAGAGAAAGGCAGTTCCTGA

Moreover, the expression of PD-related genes, including SNCA, LRRK2, cyclin G associated kinase (GAK), PINK1, ubiquitin C-terminal hydrolase L1 (UCHL1), phospholipase A2 Group VI (PLA2G6), ATP13A2, F-box protein 7 (FBXO7), and HtrA serine peptidase 2 (HTRA2), was also examined by quantitative PCR using the primers listed in Table 2.

**TABLE 2:** Quantitative PCR Primer sequence for PD-related gene.

Primer name	Primer sequence(5′-3′)
SNCA-F	CCGAGACATTCACCTGCCAAGT
SNCA-R	TGTTCCCAATTGTGTGCCAAAGTG
LRRK2-F	ATCAACCAAGGCTCACCATTCCAA
LRRK2-R	TCTCCTTCATAGGCTGCTCGGTAA
GAK-F	CTTCCTGTGCTTCTGCCGTCTC
GAK-R	CGCTCCTCTGCTTGCTGAACA
PINK1-F	TGTGTATGAAGCCACCATGCCTAC
PINK1-R	CTGGAGGAACCTGCCGAGATGT
UCHL1-F	ACCGAGCGTGAGCAAGGAGAA
UCHL1-R	TAAGTGCCTGGGTGTGGCTGAG
PLA2G6-F	TGCCATGACCGAGATCCATGAGTA
PLA2G6-R	ACCACCATCTTGCCCAGTTCCT
ATP13A2-F	ACCGACTTCGCCCAGATGCT
ATP13A2-R	TGCGGCTTCAGTAGGTTCCTCAT
FBXO7-F	CCTGCCATCGTCAACTCACACC
FBXO7-R	AAGCGTGGTCTCAGTGGAGGAA
HTRA2-F	TCTGCGGCCTGGTGATGTGAT
HTRA2-R	GAGCAGGAGCCTCATACTCTTGGT

### Immunohistochemistry and Immunofluorescence Staining

Four weeks after 6-OHDA injection, the rats were sacrificed and transcardially perfused using 4% paraformaldehyde (Sigma-Aldrich, China) in phosphate-buffered saline (PBS). Series of 40 μm thick striatal and SNc sections were obtained using a vibratome (Leica, VT1000S, Germany). Sections were immersed in 3% H_2_O_2_ for 30 min to inhibit endogenous peroxidase activity, and then permeabilized using 0.1% PBS-Triton for 10 min. For staining, the sections were blocked for 2 h with 5% fetal bovine serum (FBS; Thermo Fisher Scientific), and then incubated with primary antibody against TH (ab112; Abcam) overnight at 4°C. Subsequently, an Alexa Fluor 488 conjugated donkey anti-Rabbit IgG (H+L) secondary antibody was applied to the sections and incubated for 2–3 h at room temperature. The sections were then washed with PBS for three times, each for 5 min. Finally, the sections were attached to glass slides for visualization using Leica DMi8 microscope (Leica, Wetzlar, Germany).

Immunofluorescence staining for neural stem cells were performed in 24-well plate with cells grew on cover glass slide. Cells were fixed in cold 100% methanol for 5 min, washed with PBS, then blocked in PBS containing 10% FCS for 1 h at room temperature. The rabbit anti- paired box 6 (Pax6, 2 μg/ml, 42-6600, Thermo Fisher Scientific) and anti- SRY-related HMG-box 2 (Sox2, 1:200 dilution, PA1-16968, Thermo Fisher Scientific) polyclonal primary antibodies were used to incubate the cells at room temperature for 2 h. After washing with PBS, secondary antibodies (Alexa Fluor 488 conjugated goat anti-rabbit IgG and Alexa Fluor 594 conjugated donkey anti-rabbit IgG) were applied at 2 μg/ml for 1 h at room temperature. Finally, the cells were incubated with 10 mg/mL 4′,6-diamidino-2-phenylindole (DAPI) and observed under a fluorescence microscope.

### Western Blotting

The cell samples of NSCs transfected with mimic-control or mimic-23b-3p were collected from three independent experiment (*N* = 3), each with three repeats. The mixed protein samples of three repeats were lysed in RIPA buffer and blotted using mouse anti-SNCA monoclonal antibody (Invitrogen, Product # AHB0261) for detection of SNCA protein expression. Briefly, cell extracts (25 μg lysate) were electrophoresed using 12% Bis-Tris gel. The resolved proteins were then transferred onto a nitrocellulose membrane, washed with 1X PBS twice and probed with the primary antibody (1 μg/ml) for 2 h in room temperature. Subsequently, the membrane was incubated with HRP-conjugated Goat anti-Mouse IgG secondary antibody (Invitrogen, Product # A28177, 1:2000 dilution) for 1 h in room temperature. Finally, the expression of SNCA was detected by chemiluminescence using an ECL chemiluminescent substrate kit (Abcam, ab65623) according to manufacturer’s protocol. The densitometric analysis was performed using ImageJ software and normalized to the density of β-actin from the same blot.

### miRNA Target Assay

The untranslated region (UTR) of SNCA was amplified from the human genome and subcloned into pMiRluc to generate the SNCA-UTR-luciferase (firefly) expression plasmid pMiRluc-SNCA. A plasmid harboring a mutant in the SNCA-UTR binding site was generated using a site-directed mutagenesis kit (Stratagene) with PCR primers containing the mutant site, and the resulting plasmid was designated pMiRluc-SNCAM. The primers used for PCR amplification were tSNCA -F, GCTAGCAGTTTATAAAAGGAGAAAAAGGTATTCCTATA TATTGGGCGCTGGTGAG, and tSNCA-R, CGCCCAATATAT AGGAATACCTTTTTCTCCTTTTATAAACTGCTAGCATGTC TAG.

For the miRNA target assay, HEK293 cells were transfected with pMiRluc-SNCA or pMiRluc-SNCAM together with a mimic-control or mimic-23b-3p. The plasmid containing the Renilla luciferase gene was also transfected as the internal control. After overnight incubation, the relative firefly luciferase activity from three independent experiments (*N* = 3) was measured and normalized to the Renilla activity according to the manufacturer’s protocol of the Dual-Luciferase Reporter Assay System (E1910, Promega).

### Statistical Analysis

All data are expressed as mean + S.E.M. Statistical comparisons between two groups were performed using either the parametric Student *t*-test or the non-parametric Mann–Whitney *U*-test after assessing for normality with the Shapiro–Wilk test. Statistical comparisons among more than two groups were performed using either the parametric ANOVA or the non-parametric Kruskal–Wallis test, as appropriate. Bonferroni adjustments were used to correct for multiple comparisons. Statistical significance was set at a value of *p* < 0.05. Statistical analyses were performed using SPSS 23.0 (IBM, Armonk, NY, United States). Graphs were drawn using GraphPad Prism 8.00 (GraphPad Software, San Diego, CA, United States).

## Results

### Circulating Exosome Purification From the Plasma of PD Patients

**FIGURE 1:**
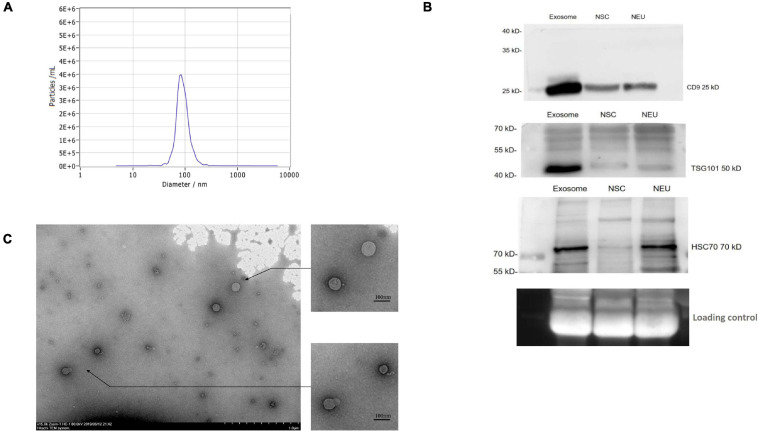
Purification and identification of circulating exosomes from PD patient plasma. **(A)** Exosomes were extracted from patient plasma and characterized by a NanoSight NS300. The figure shows the representative NanoSight data obtained from patient plasma samples. **(B)** The surface markers of exosomes were identified by Western blotting using antibodies against CD9, TSG101, and HSC70. The figure shows the representative Western blotting results using the samples of circulating exosome, NSCs and NSC derived neuron (NEU). **(C)** The isolated particles from patient plasma were observed under TEM, and the figure shows the representative morphology of the isolated particles. NSC, neural stem cell; NEU, neuron. Scale bar, 1.0 μm in original photo and 100 nm in enlarged photo.

To purify circulating exosomes from PD patient plasma, a membrane affinity spin column method was employed due to the fast procedure and high yield (Supplementary Figure 1). As determined by NTA, the concentration of the isolated particles with a diameter of 85.7 nm was 3.4E+6/ml, which accounted for 98.5% of the isolated particle population (Figure 1A). Furthermore, the general surface markers of exosomes were examined by Western blotting using antibodies against CD9, TSG101, and HSC70. The results from Western blotting showed strong bands for these surface markers (Figure 1B), confirming the general exosomal characteristics. In addition, these surface markers on the exosomes released from NSCs and NSC-derived mature neurons (NEU) were investigated. Compared to patient plasma sample, the NSC- and NEU-derived exosomes express similar surface marker proteins, but with less expression (Figure 1B). Finally, the isolated particles were observed by TEM, and these particles exhibited a round shape with a membrane structure (Figure 1C). These data collectively confirmed the successful purification of exosomes from PD patient plasma.

### miRNA Profiles in the Plasma and Circulating Exosomes of PD Patients

**FIGURE 2:**
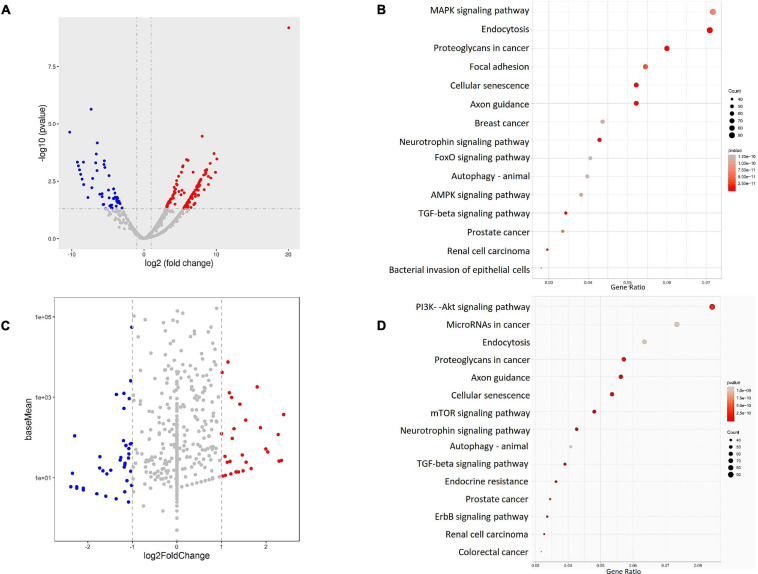
The miRNA profiles of the PD patient (*N* = 22) and healthy volunteers (*N* = 9) plasma **(A,B)** and exosomes **(C,D)** were identified by RNA sequencing, and the results were visualized by **(A,C)** a volcano plot, which shows the differentially expressed miRNAs. The target genes of the identified miRNAs were analyzed by **(B,D)** KEGG pathway enrichment to identify the possible downstream signaling pathways.

To verify the miRNA profiles in the plasma and circulating exosomes of PD patients, peripheral blood samples of PD patients (*N* = 22) and healthy volunteers (*N* = 9) were collected and analyzed for small RNAs, mainly miRNAs, profiles by HiSeq sequencing. Differentially expressed miRNAs were identified in both plasma and circulating exosomes between PD patients and healthy volunteers. In summary, 167 differentially expressed miRNAs were identified in plasma, and 107 miRNAs were identified in circulating exosomes. Among these miRNAs, 112 were upregulated, ranging from 3.08- to 20.04-fold in the plasma (Figure 2A), and 35 were upregulated in circulating exosomes, ranging from 1.001- to 2.396-fold (Figure 2C). In addition, 55 miRNAs in the plasma and 72 miRNAs in the circulating exosome were downregulated with a range from –3.028 to –10.279-fold and –1.016 to –4.481-fold, respectively. Interestingly, the functional enrichment GO (Supplementary Figures 2, 3) and KEGG (Figures 2B,D) analyses revealed that the plasma and exosome miRNA-regulated target genes were enriched in similar pathways, including the MAPK-PI3K signaling pathway.

Wnt signaling pathway, endocytosis, focal adhesion, axon guidance, neurotrophin signaling pathway, TGF beta signaling pathway, and in some cancer cell line profiles. These data confirm that the miRNAs in both plasma and exosomes of PD patients share similar regulatory networks and that both sampling methods have similar power for PD disease prediction. Notably, the KEGG analysis also revealed the regulatory roles of these miRNA-regulated target genes in transcription factor or activator activity and promoter proximal region DNA binding (Figures 2B,D), indicating a possible transcriptional regulation of downstream genes.

**FIGURE 3:**
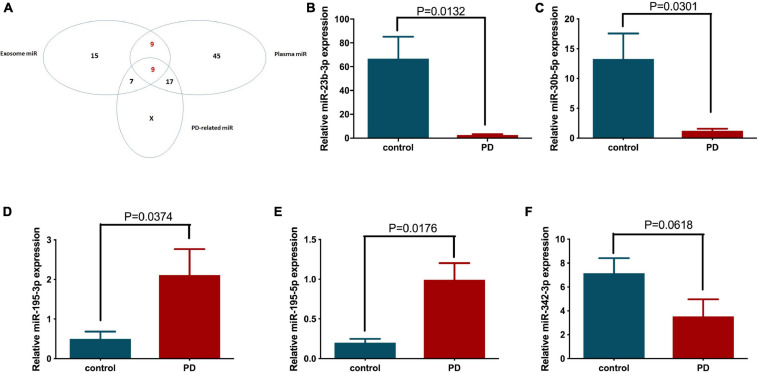
The differentially expressed miRNAs between healthy volunteer and PD patient plasma and circulating exosomes were classified, and their expression patterns in patient plasma were further confirmed by quantitative PCR. **(A)** The Venn diagram shows the relationships between the differentially expressed plasma miRNAs, circulating exosomal miRNAs and the bioinformatics predicted PD-related miRNAs. The expression levels of **(B)** miR-23b-3p, **(C)** miR-30b-5p, **(D)** miR-342-3p, **(E)** miR-195-3p, and **(F)** miR-195-5p in the individual patient (*N* = 5) and healthy volunteer (*N* = 7) plasma were examined by quantitative PCR.

Therefore, we further investigated the transcriptional regulation of these miRNAs on PD-related genes. The top 20 upregulated and downregulated differentially expressed miRNAs from plasma and exosomes (using two extraction kits, designated Qiagen and BIOG) were selected (Table 3) and analyzed for their possible regulatory roles on PD-related genes, including SNCA, LRRK2, GAK, PINK1, UCHL1, PLA2G6, ATP13A2, FBXO7 and HTRA2, using microRNA.org and TargetScan (Table 4). As expected, the results confirm that these differentially expressed miRNAs have potential targeting sites on the UTRs of various PD-related genes and thus may be involved in the pathogenesis of PD via regulation of PD-related genes. Next, we identified 18 miRNAs that were appeared both in the lists of differentially expressed plasma and exosome miRNAs, as shown in Table 3 (Table 5). These 18 miRNAs were further compared with PD-related miRNAs in Table 4, and 9 miRNAs were selected for use in subsequent analysis (Figure 3A).

**TABLE 3:** Top 20 upregulated and downregulated miRNAs (based on Log2Fold-Change) in the exosome and plasma of PD patients.

Upregulated miRNAs (Log2Fold-Change level)			Downregulated miRNAs (Log2Fold-Change level)		
	
Exosome miR	Plasma miR-Qiagen	Plasma miR-BIOG	Exosome miR	Plasma miR-Qiagen	Plasma miR-BIOG
miR-184 (2.3965)	miR-122-3p (20.0451)	miR-7704 (4.6514)	hsa-miR-141-3p (–4.4820)	hsa-miR-3163 (–10.2798)	hsa-miR-1301-3p (–2.2151)
miR-30b-3p (2.3478)	miR-30b-5p (10.0961)	miR-363-3p (3.0793)	hsa-miR-130b-5p (–4.1465)	hsa-miR-6894-3p (–9.2157)	hsa-miR-106a-5p (–1.9588)
hsa-miR-195-5p (2.2925)	hsa-miR-455-5p (9.8985)	hsa-miR-185-5p (2.7438)	hsa-miR-3143 (–3.4884)	hsa-miR-128-2-5p (–9.2157)	hsa-miR-193a-5p (–1.7699)
hsa-miR-4661-5p (2.2925)	hsa-miR-654-5p (9.7007)	hsa-miR-93-5p (1.9862)	hsa-miR-619-5p (–3.4694)	hsa-miR-1262 (–9.0782)	hsa-miR-6734-5p (–1.7663)
hsa-miR-342-3p (2.2707)	hsa-miR-195-5p (9.2498)	hsa-miR-486-3p (1.8666)	hsa-miR-6515-5p (–3.0784)	hsa-miR-1293 (–8.9262)	hsa-miR-342-3p (–1.7663)
hsa-miR-23b-5p (2.0412)	hsa-miR-493-5p (9.1623)	hsa-miR-619-5p (1.7869)	hsa-miR-6516-5p (–2.9112)	hsa-miR-3154 (–8.7562)	hsa-miR-3177-3p (–1.3311)
hsa-miR-708-3p (1.9956)	hsa-miR-31-5p (8.9231)	hsa-miR-146b-5p (1.7453)	hsa-miR-1287-5p (-2.8198)	hsa-miR-4658 (–8.5636)	hsa-miR-4433b-5p (–1.3311)
hsa-miR-129-5p (1.8732)	hsa-miR-145-5p (8.9069)	hsa-miR-769-5p (1.7025)	hsa-miR-5695 (–2.5052)	hsa-miR-885-3p (–8.3740)	hsa-miR-326 (–1.3311)
hsa-miR-150-5p (1.8022)	hsa-miR-339-5p (8.6858)	hsa-miR-4488 (1.7025)	hsa-miR-130b-3p (–2.5026)	hsa-miR-4700-5p (–8.3412)	hsa-miR-629-5p (–1.1780)
hsa-miR-181c-5p (1.6675)	hsa-miR-340-3p (8.6736)	hsa-miR-19b-3p (1.3612)	hsa-miR-3682-3p (–2.3832)	hsa-miR-10394-3p (–8.3412)	hsa-miR-423-5p (–1.1593)
hsa-miR-130 (1.6675)	hsa-miR-24-2-5p (8.5525)	hsa-miR-10400-5p (1.2468)	hsa-miR-1277-5p (–2.3832)	hsa-miR-4731-5p (–8.3412)	hsa-miR-10a-5p (–1.1567)
hsa-miR-199b-5p (1.5552)	hsa-let-7a-3p (8.5151)	hsa-miR-126-5p (1.2468)	hsa-miR-135a-5p (–2.3487)	hsa-miR-483-3p (–8.3412)	hsa-miR-139-3p (–1.1379)
hsa-miR-95-3p (1.5426)	hsa-miR-200c-3p (8.4858)	hsa-miR-20b-5p (1.2464)	hsa-miR-4433b-3p (–2.2960)	hsa-miR-6881-3p (–8.3412)	hsa-miR-222-3p (–1.1092)
hsa-miR-338-3p (1.4824)	hsa-miR-218-5p (8.3401)	hsa-miR-186-5p (1.2024)	hsa-miR-4433b-3p (–2.2960)	hsa-miR-6881-3p (–8.3412)	hsa-miR-222-3p (–1.1092)
hsa-miR-7849-3p (1.4824)	hsa-miR-654-3p (8.2740)	hsa-let-7a-3p (1.1226)	hsa-miR-324-3p (–2.2501)	hsa-miR-193b-5p (–7.3206)	hsa-miR-937-3p (–1.0549)
hsa-miR-197-3p (1.4664)	hsa-miR-23b-3p (8.0521)	hsa-miR-497-5p (–1.0549)	hsa-miR-4489 (–2.1037)	hsa-miR-449 (–7.2148)	hsa-miR-195-3p (–1.0549)
hsa-miR-155-5p (1.4142)	hsa-miR-374b-5p (7.8791)	hsa-miR-184 (–1.0549)	hsa-miR-487a-5p (–2.1037)	hsa-miR-3130-3p (–7.0994)	hsa-miR-184 (–1.0549)
hsa-miR-1273h-5p (1.3801)	hsa-miR-433-3p (7.8752)	hsa-miR-195-3p (–1.0549)	hsa-miR-643 (–2.1037)	hsa-miR-4657 (–6.6779)	hsa-miR-497-5p (–1.0549)
hsa-miR-431-3p (1.3262)	hsa-miR-9-5p (7.7546)	hsa-miR-937-3p (–1.0549)	hsa-miR-29c-3p (–2.1037)	hsa-miR-1228-5p (–6.6016)	hsa-let-7a-3p (1.1226)
hsa-miR-30c-2-3p (1.2788)	hsa-miR-32-5p (7.7259)	hsa-miR-224-5p (–1.0549)	miR-6826-5p (–1.8013)	miR-4732-5p (–6.4978)	miR-186-5p (1.2024)

**TABLE 4:** The identified miRNAs that regulate PD-related genes.

Gene name	Target microRNA	Gene name	Target microRNA
ATP13A2	miR-24	DJ1	miR-128
	miR-122	GIGYF2	miR-19
	miR-195		miR-30
	miR-199		miR-31
	miR-433		miR-122
	miR-497		miR-128
GAK PARK17	miR-20		miR-150
	miR-93		miR-186
	miR-106		miR-197
	miR-455		
HTRA2	miR-20	LRRK2	miR-30
	miR-93		miR-130
	miR-106		miR-141
	miR-145	PARK1 (SNCA)	miR-23b
	miR-340		miR-129
	miR-374		miR-342
	miR-497		miR-374b
	miR-708	PLA2G6	miR-20b
PINK1	miR-24		miR-93
	miR-199		miR-106a
	miR-433		miR-195
	miR-497		miR-324
UCHL1	miR-9		
	miR-130b		

**TABLE 5:** Selected panel of the miRNAs for functional analysis.

Panel	
hsa-miR-184	hsa-miR-4433b-3p
hsa-miR-30b-3p	hsa-miR-4433b-5p
hsa-miR-30b-5p	hsa-miR-130b-3p
hsa-miR-654-5p	hsa-miR-130b-5p
hsa-miR-654-3p	hsa-miR-193b-5p
hsa-miR-342-3p	hsa-miR-193a-5p
hsa-miR-23b-5p	hsa-miR-195-5p
hsa-miR-23b-3p	hsa-miR-195-3p
hsa-miR-619-5p	hsa-let-7a-3p

### Validation of Altered miRNA Expression in PD Patients and Disease Models

The nine selected miRNAs were further validated in the plasma of individual healthy volunteer (*N* = 7) and PD patients (*N* = 5) by quantitative PCR to determine the expression levels of these miRNAs. Five of 9 selected miRNAs showed altered expression in the PD patient group compared to the age-matched control group. Specifically, the expression levels of miR-23b-3p and miR-30b-5p were reduced significantly by 96.24 and 90.77%, respectively (Figures 3B,C). In contrast, miR-195-3p and miR-195-5p showed significantly elevated expression in PD patient plasma, with 4.24- and 4.98-fold increases, respectively, compared to the control group (Figures 3D,E). In addition, the expression of miR-342-3p also decreased in PD patients, although the difference was not statistically significant (*p* = 0.0618) due to the limited sample number (Figure 3F).

We next investigated miRNA expression in both *in vitro* and animal models of PD. First, NSCs were characterized by immunofluorescence staining of two neuron markers, Pax6 and Sox2. Indeed, the cells exhibited high expression levels of Pax6 and Sox2, which correlated with DAPI staining (Supplementary Figure 4). Moreover, the cells formed a rosette structure, which was known to be a critically important structure during neural development.

**FIGURE 4:**
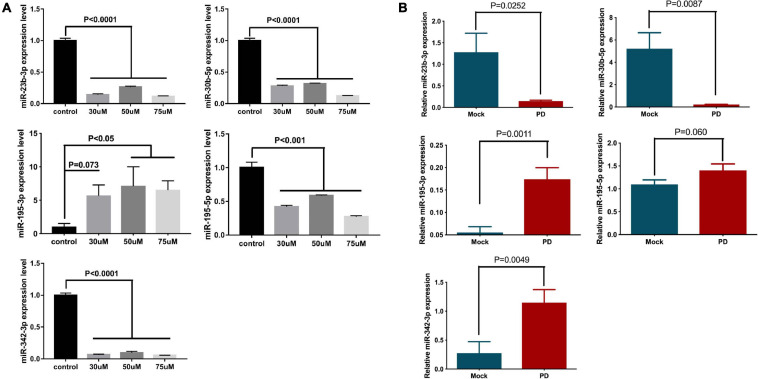
The expression levels of miR-23b-3p, miR-30b-5p, miR-342-3p, miR-195-3p, and miR-195-5p in the *in vitro* and *in vivo* Parkinsonism models. **(A)** NSCs were treated with 30, 50, and 75 μM 6-OHDA for 24 h, and the expression of miR-23b-3p, miR-30b-5p, miR-342-3p, miR-195-3p, and miR-195-5p was measured. The data are collected from three independent experiments (*N* = 3) and expressed as mean + SEM. **(B)** The expression levels of miR-23b-3p, miR-30b-5p, miR-342-3p, miR-195-3p, and miR-195-5p were tested in the 6-OHDA-induced rat Parkinsonism model group (*N* = 8) and control mock injection group (*N* = 6).

To generate the model, NSCs were treated with 6-OHDA, a specific neurotoxin, and examined for miRNA expression. As expected, the expression levels of miR-23b-3p, miR-30b-5p, and miR-342-3p were significantly decreased by at least 68% upon 30, 50, and 75 μM 6-OHDA treatment, compared to the vehicle control (Figure 4A). The expression of miR-195-3p was significantly increased by 3.9- to 10.39-fold under the same treatment conditions (Figure 4A). However, the expression of miR-195-5p in the 6-OHDA-treated cells was decreased, which was contradictory to its expression in the PD patients’ plasma.

The expression of miRNA was further investigated in a rat model of PD. The striatum of Wistar rats was injected with 6-OHDA to generate parkinsonism symptoms. Damage to the striatum was confirmed by immunohistochemistry using an anti-TH antibody (Supplementary Figure 5A), and behavioral disorders in these rats were also validated by cylinder tests, stepping tests and bilateral tactile stimulation assays (Supplementary Figure 5B). Similar to previous results, the expression levels of miR-23b-3p and miR-30b-5p in the plasma of the 6-OHDA-injected rats were significantly decreased, and the expression level of miR-195-3p was increased compared to the level of the control group (Figure 4B). Notably, the expression of miR-342-3p was increased, which is opposite to previous cell and patient studies. Additionally, the expression of miR-195-5p showed no significant difference between 6-OHDA-injected and control rats.

### miR-23b-3p Directly Targets the Alpha-Synuclein Gene

Since miR-23b-3p, miR-30b-5p, and miR-195-3p demonstrated consistent expression patterns in the cell and rat models of PD, as well as patient plasma, their functions in the regulation of PD-related genes were further analyzed.

**FIGURE 5:**
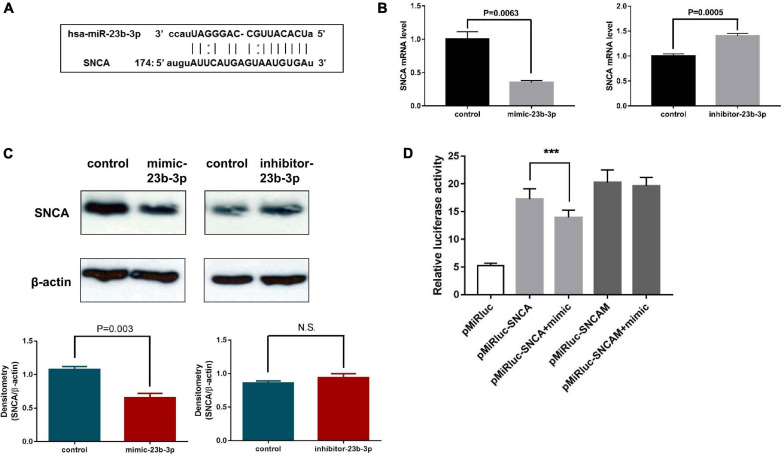
miR-23b-3p, miR-30b-5p, and miR-195-3p coordinately regulate downstream PD-related target genes in NSCs. **(A)** Diagram showing the direct targeting of miR-23b-3p on the UTR of SNCA. **(B)** The mRNA levels of SNCA were examined in NSCs transfected with miR-23b-3p mimic (mimic-23b-3p) or inhibitor (inhibitor-23b-3p) by quantitative PCR. **(C)** The protein level of SNCA was examined in NSCs transfected with miR-23b-3p mimic (mimic-23b-3p) or miR-23b-3p inhibitor (inhibitor-23b-3p) by Western blotting using antibody against SNCA. **(D)** The luciferase activity of HEK293 cells transfected with pMiRluc-SNCA or pMiRluc-SNCAM (mutation in the miRNA targeting site) together with mimic-control (pMiRluc-SNCA) or mimic-23b-3p (pMiRluc-SNCA+mimic). After incubation overnight, the relative luciferase activity was measured and normalized to the Renilla activity. ***means statistically *P* ≤ 0.001.

As miR-23b-3p was predicted to directly target the 3′-UTR of SNCA mRNA using computational miRNA target prediction tools (Figure 5A), the possible regulation of miR-23b-3p on the SNCA gene was investigated by experimental approaches. The chemically synthesized miR-23b-3p mimic and inhibitor were used to overexpress and inhibit miRNA expression in human NSCs, and the expression levels of SNCA mRNA were detected by quantitative PCR. The effects of the miR-23b-3p mimic and inhibitor were confirmed (Supplementary Figure 6A), and as expected, the mRNA level of SNCA was decreased by 65% in miR-23b-3p mimic-transfected cells but increased by 1.4-fold in inhibitor-transfected cells (Figure 5B). Moreover, the protein level of SNCA was decreased significantly in miR-23b-3p mimic transfected cells and remained unchanged in the miR-23b-3p inhibitor transfected cells as quantified by densitometric analysis of the Western blots (Figure 5C). In addition, the predicted target site of SNCA was cloned into a dual-luciferase miRNA target expression vector and tested for luciferase activity in HEK293T cells transfected with miR-23b-3p mimic. Not surprisingly, transfection of the miR-23b-3p mimic significantly inhibited luciferase activity in pMiRluc-SNCA-transfected HEK293T cells but not in mutant pMiRluc-SNCAM-transfected cells (Figure 5D).

### Modulation of PD Genes by miRNAs

**FIGURE 6:**
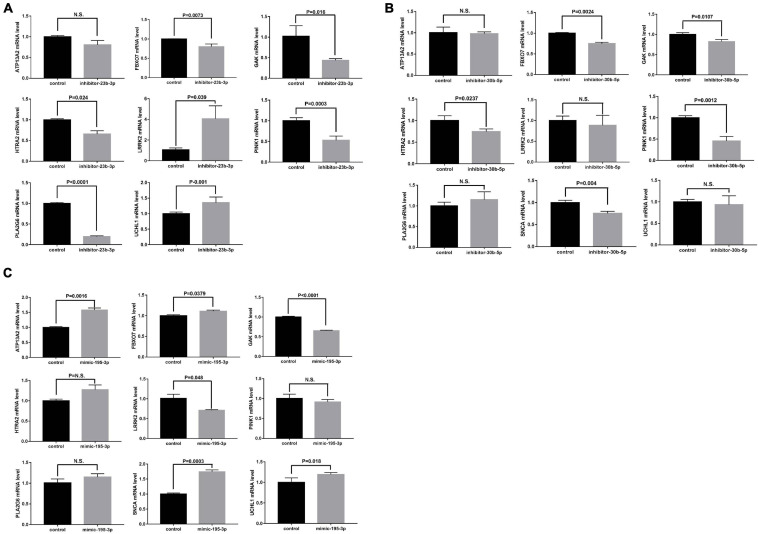
NSCs were transfected with **(A)** miR-23b-3p inhibitor (inhibitor-23b-3p), **(B)** miR-30b-5p inhibitor (inhibitor-30b-5p), or **(C)** miR-195-3p (mimic-195-3p) and tested for the expression of PD-related genes, including ATP13A2, FBXO7, GAK, HTRA2, LRRK2, PINK1, PLA2G6, SNCA, and UCHL1 All data are collected from three independent experiments (*N* = 3) and expressed as mean + SEM.

Moreover, the possible miRNA-mediated indirect regulation of PD-related genes, including SNCA, LRRK2, GAK, PINK1, UCHL1, PLA2G6, ATP13A2, FBXO7, and HTRA2, was determined in miRNA mimic- or inhibitor-transfected neural stem cells. As miR-23b-3p was decreased in the patient plasma, the miR-23b-3p inhibitor was used to suppress miRNA expression in the cells. Interestingly, the expression of LRRK2 and UCHL1 was significantly increased, while the expression of FBXO7, GAK, HTRA2, PINK1, and PLA2G6 was decreased by 20–80.3% compared to that in control cells (Figure 6A). However, the expression of ATP13A2 was unchanged by miR-23b-3p inhibitor transfection (Figure 6A). Similarly, the miR-30b-5p inhibitor was transfected into neural stem cells to mimic the decreased expression of miR-30b-5p in PD patient plasma (Supplementary Figure 6B). Similar to the effects of miR-23b-3p, the quantitative PCR results revealed that the expression of GAK and PINK1, in addition to that of FBXO7 and HTRA2, was decreased significantly in miR-30b-5p inhibitor-transfected cells (Figure 6B). However, the expression of SNCA was slightly decreased in the cells transfected with miR-30b-5p inhibitor. Additionally, the expression of ATP13A2, LRRK2, PLA2G6, and UCLH1 was unchanged (Figure 6B). Moreover, as miR-195-3p expression was elevated in PD patient plasma, the effects of miR-195-3p overexpression on PD-related genes were investigated using a miRNA mimic (Supplementary Figure 6C). Overexpression of miR-195-3p led to decreased expression of LRRK2 and GAK (Figure 6C). In contrast, increased expression of UCHL1 and SNCA was observed in the miR-195-3p mimic-transfected cells, similar to the effects of the miR-23b-3p inhibitor. The expression of HTRA2, PINK1, and PLA2G6 was not changed, while the expression of FBXO7 was slightly increased in miR-30b-5p inhibitor transfected cells (Figure 6C). These data confirm that the identified miRNAs have differential regulatory roles in PD-related gene expression in neural cells.

## Discussion

Circulating miRNAs are highly stable in extracellular vehicles and have been implicated in many disease states by modulating disease-associated genes (Korabecna et al., 2013; Andersen et al., 2014; Leggio et al., 2017). Although the mechanism regulating the selective secretion of circulating miRNAs remains unclear, circulating miRNAs have been demonstrated to have potential value in the diagnosis, prognosis and treatment of various diseases (Cho, 2011; Chen H. et al., 2014; Turchinovich and Cho, 2014; Turchinovich et al., 2015). In addition, the intake of circulating miRNAs by recipient cells has been demonstrated to have biological functions (Kosaka et al., 2013). Currently, the circulating miRNA pattern in PD patients has not yet been well defined. Several studies have analyzed circulating miRNA levels in patients with PD compared with healthy controls. For instance, the dysregulation of miRNAs has been identified in the plasma of idiopathic and genetic PD patients with relative bigger sample size (Ravanidis et al., 2020a, b). However, the results of these previous studies were based on a single studied sample type, such as plasma, serum, or exosomes (Khoo et al., 2012; Dong et al., 2016; Chen L. et al., 2018). In the present study, we used next-generation sequencing to simultaneously analyze miRNA profiles in both plasma and circulating exosomes of patients with PD, and the overlapping differentially expressed miRNAs were selected for further study. As expected, the overlapping miRNAs comprised a small fraction of the total differentially expressed miRNAs, with 45% in exosomes and 22.5% in plasma (Figure 3A), implying the drawback of using a single-source sample. However, interestingly, GO function and KEGG pathway analysis revealed that these plasma and exosome differentially expressed miRNA-regulated target genes were enriched in similar biological processes and pathways, such as the MAPK-PI3K signaling pathway, Wnt signaling pathway, axon guidance, neurotrophin signaling pathway, and TGF beta signaling pathway. Since these processes and pathways have been previously reported to be involved in PD pathogenesis by regulating neural functions (Nataraj et al., 2017; Marchetti, 2018; Munoz et al., 2020), our findings suggest that the circulating miRNAs that coexist in plasma and circulating exosomes may contribute to the etiology of PD, further confirming the unique value of miRNAs in PD diagnosis and therapy development. Combined with target miRNA prediction using PD-related genes (Table 4), 9 potential PD-related circulating miRNAs that coexist in plasma and exosomes were selected for further analysis. Among these, 5 miRNAs were validated by quantitative PCR in individual patient samples. More importantly, the similar expression patterns of miR-23b-3p, miR-30b-5p, and miR-195-3p were reconfirmed in neural cells and a rat model of PD. In line with our results, upregulated miR-30b-5p expression has been demonstrated in L-dopa-treated PD-diagnosed patients (Serafin et al., 2015). Suppression of miR-342-3p has been shown to prevent DA neuron loss in mice (Wu et al., 2019). Moreover, miR-195 was previously identified as a biomarker for PD in a panel of five serum miRNAs (Ding et al., 2016). Notably, the expression of some miRNAs between patient plasma, NSCs and 6-OHDA injected rat model was contradict. This is probably due to the small sample size in the patient group, the difference between *in vivo* and *in vitro* study, as well as the difference between rat and human. However, only the ones that show consistent expression fashion in all studies were selected as PD-related miRNAs.

To our knowledge, we are the first to identify miR-23b-3p as a novel PD-related circulating miRNA in PD patients. Previously, miR-23b had been demonstrated to relieve neuroinflammation and brain injury-related or hypoxia-induced neuronal apoptosis, which may be associated with PD pathogenesis (Chen Q. et al., 2014; Hu et al., 2019). Although miR-23b has been proposed as a biomarker for multiple sclerosis (Martinez and Peplow, 2020), the role of miR-23b-3p in PD is still unknown. SNCA is a well-defined pathological hallmark of PD, and aberrant soluble oligomeric conformations, termed protofibrils, are generally thought to disrupt cell homeostasis and neuronal death. Two miRNAs, miR-7 and miR-153, have been shown to repress SNCA expression posttranscriptionally (Junn et al., 2009; Doxakis, 2010). However, alterations in the expression of these miRNAs have yet to be determined in PD patients. In this study, we established that miR-23b-3p directly targets the 3′-UTR of SNCA, leading to a decrease in gene expression. Moreover, our results demonstrated that the inhibition of miR-23b-3p alone was sufficient to significantly increase SNCA expression, confirming its potential as a therapeutic target. In addition to SNCA, miR-23b-3p may also indirectly regulate the expression of other PD-related genes (Figure 5D). Interestingly, these PD-related genes have been reported to interact with SNCA by influencing its expression or function. For instance, the protective effects of PINK1 against SNCA-induced neurodegeneration were shown previously (Oliveras-Salva et al., 2014; Liu et al., 2017). Moreover, GAK could modify SNCA expression levels and affect susceptibility to PD (Tseng et al., 2013). Taken together, the findings led us to speculate that miR-23b-3p may directly target SNCA expression or indirectly influence the function of SNCA through regulation of PD-related genes to mediate PD pathogenesis.

In a previous study, miR-30b was proven to be a direct regulator of SNCA and protects dopaminergic neuroblastoma cells from MPP(+)-induced neurotoxicity (Shen et al., 2020). These data suggest that the level of SNCA in PD may be regulated by multifaceted mechanisms. Similar to miR-23b-3p, our results showed that inhibiting the activity of miR-30b-5p could significantly decrease GAK and PINK1 levels, indicating their synergistic effects in modulating PD-related gene expression. In addition, miR-30b-5p has been reported to be the central regulator that is dysregulated in four neurodegenerative diseases (Brennan et al., 2019). Therefore, miR-30b-5p may not serve as a unique biomarker for PD. The functions of miR-195 are mainly discovered in the regulation of cell proliferation and apoptosis, which play important roles in degenerative diseases (Chen L. P. et al., 2018; Luo et al., 2019). A previous study has revealed that miR-195 triggered neuroinflammation, a central pathological change in the progression of PD, in a Rho-associated kinase 1-dependent manner (Ren et al., 2019). Consistently, our results confirmed that miR-195-3p was involved in the regulation of PD-related genes, such as GAK, UCHL1, and SNCA, in a fashion similar to that of the miR-23b inhibitor. However, LRRK2 expression was decreased in the miR-195-3p mimic-transfected cells, in contrast to the effects of the miR-23b inhibitor. These findings further strengthen the idea that, in addition to synergistic patterns, these miRNAs exhibit various patterns in the regulation of PD-related genes, forming a multifactorial regulatory network.

In summary, we identified novel PD-related circulating miRNAs in both the plasma and exosomes of PD patients. More importantly, our findings suggest that these aberrantly expressed miRNAs directly or indirectly regulate the expression of PD-related pathogenic genes. The interactions between these miRNAs, the downstream PD pathogenic hallmark SNCA and other PD-related genes form a complex regulatory network in PD pathogenesis. Therefore, these miRNAs may serve as potential therapeutic targets for treating PD.

## Data Availability Statement

The datasets presented in this study can be found in online repositories. The names of the repository/repositories and accession number(s) can be found below: Sequence Read Archive, accession numbers SAMN19372448, SAMN19372449, SAMN19372450, SAMN19372451, and SAMN19372452.

## Ethics Statement

The studies involving human participants were reviewed and approved by Ethic Committee of Tongji Medical School of Huazhong University of Science and Technology. The patients/participants provided their written informed consent to participate in this study. The animal study was reviewed and approved by Ethic Committee of Zhongnan Hospital of Wuhan University.

## Author Contributions

MC: experimental design, data analysis, and original draft preparation. SC: sample collection and original draft preparation. TX: sample and case data collection, and methodology. JW: data analysis and experimental design. WM and YaZ: investigation, data analysis, and drafting the figures. XL and WW: experimental design. XD, BY, WL, BS, and MW: case data collection and analysis. TL, YC, ZZ, ZM, YiZ, and JY: sample storage and transportation. JC and NX: conception and design of the study, and case data collection. JZ, LS, and JH: manuscript revisions. All authors contributed to the article and approved the submitted version.

## Conflict of Interest

The authors declare that the research was conducted in the absence of any commercial or financial relationships that could be construed as a potential conflict of interest.

## Publisher’s Note

All claims expressed in this article are solely those of the authors and do not necessarily represent those of their affiliated organizations, or those of the publisher, the editors and the reviewers. Any product that may be evaluated in this article, or claim that may be made by its manufacturer, is not guaranteed or endorsed by the publisher.

## Footnotes

1
http://www.microrna.org/microrna/home.do

2
http://pictar.mdc-berlin.de/

3
http://www.targetscan.org/

4
https://www.genome.jp/kegg/

## Supplementary Material

Supplementary Figure 1 The efficiency of the membrane affinity spin column method for exosome isolation was tested by western blotting in the Input (NSCs culture supernatant) and Flow (isolated exosomes) samples using antibodies against exosomal marker proteins, CD9, TSG101, and HSC70.

Supplementary Figures 2, 3 The miRNA profiles of the PD patient plasma (Supplementary Figure 1) and exosomes (Supplementary Figure 2) were identified by RNA sequencing, and the target genes of the identified miRNAs were analyzed by gene ontology (GO) pathway enrichment to identify the possible downstream signaling pathways.

Supplementary Figure 4 The NSCs were characterized using immunofluorescence staining with antibodies against human Pax6 and Sox2 protein. In addition, the cells were stained with DAPI for visualization of the nucleus.

Supplementary Figure 5 The rat Parkinsonism model was induced by injection of 6-OHDA into the striatum, and damage to the striatum and substantia nigra (right side) was confirmed by **(A)** immunohistochemistry staining using an anti-TH antibody. The figure shows a representative immunohistochemistry staining in the 6-OHDA injected rat brain tissue. **(B)** The motor and sensing behavior of the model rats was examined by cylinder test, stepping test, and bilateral tactile stimulation assay. The data are collected from 58 6-OHDA injected rats (*N* = 58) and expressed as mean + SEM.

Supplementary Figure 6 NSCs were transfected with **(A)** miR-23b-3p mimic (mimic-23b-3p) or inhibitor (inhibitor-23b-3p), **(B)** miR-30b-5p inhibitor (inhibitor-30b-5p), and **(C)** miR-195-3p mimic (mimic-195-3p). The effects of inhibition or overexpression of miRNAs were measured by quantitative PCR. The data are collected from three independent experiments (*N* = 3) and expressed as mean + SEM.
