# Supplementary figures and images for: Aberrant Expression of Circulating MicroRNA Leads to the Dysregulation of Alpha-Synuclein and Other Pathogenic Genes in Parkinson’s Disease
Source: Front Cell Dev Biol. 2021 Aug 23;9:695007. doi: 10.3389/fcell.2021.695007 (PMC8419519; doi:10.3389/fcell.2021.695007)

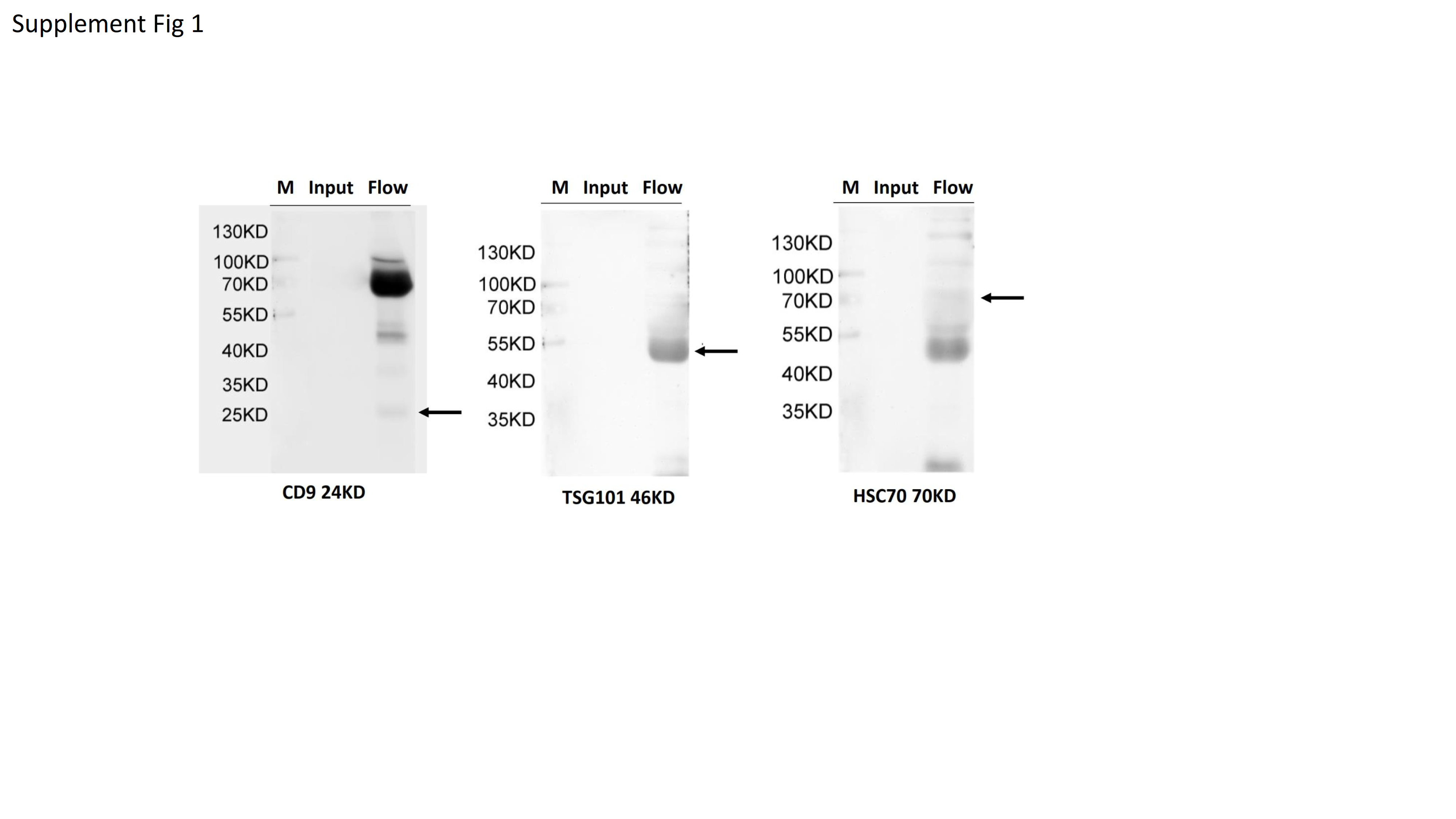

Supplement: Supplementary Figure 1 — The efficiency of the membrane affinity spin column method for exosome isolation was tested by western blotting in the Input (NSCs culture supernatant) and Flow (isolated exosomes) samples using antibodies against exosomal marker proteins, CD9, TSG101, and HSC70. [file Image_1.TIF]

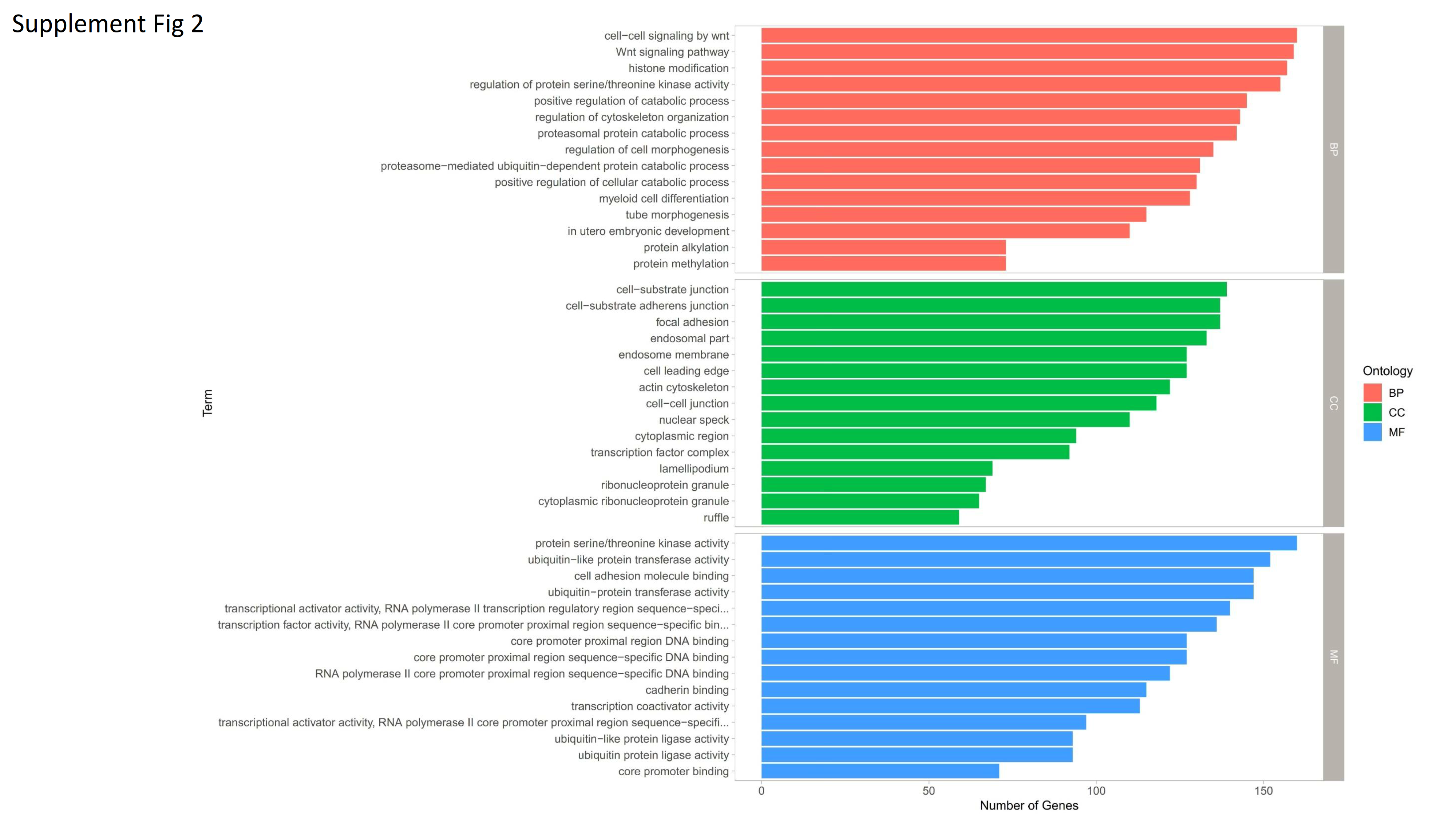

Supplement: Supplementary Figures 2, 3 — The miRNA profiles of the PD patient plasma (Supplementary Figure 1) and exosomes (Supplementary Figure 2) were identified by RNA sequencing, and the target genes of the identified miRNAs were analyzed by gene ontology (GO) pathway enrichment to identify the possible downstream signaling pathways. [file Image_2.TIF]

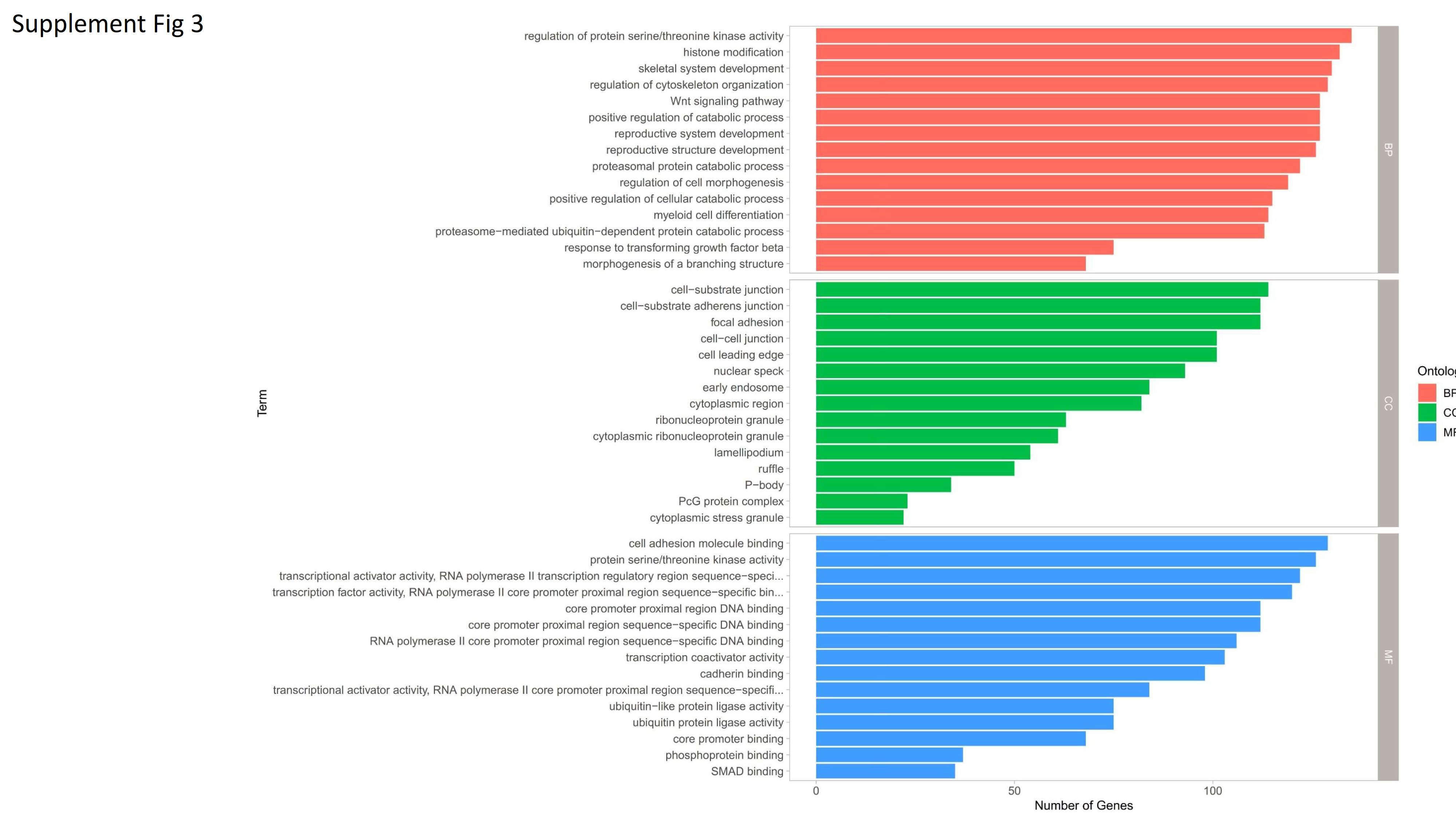

Supplement: Supplementary file 3 [file Image_3.TIF]

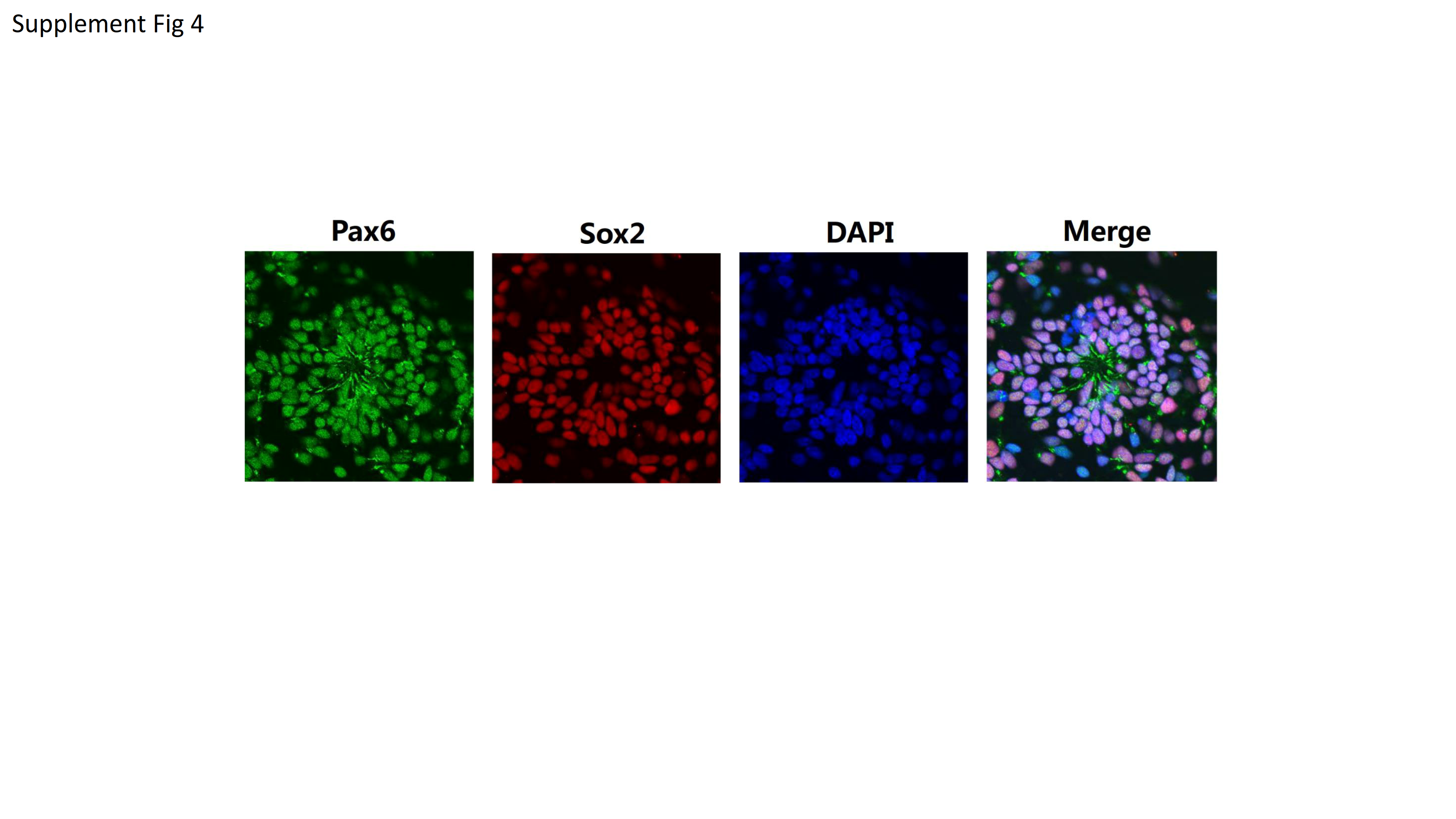

Supplement: Supplementary Figure 4 — The NSCs were characterized using immunofluorescence staining with antibodies against human Pax6 and Sox2 protein. In addition, the cells were stained with DAPI for visualization of the nucleus. [file Image_4.TIF]

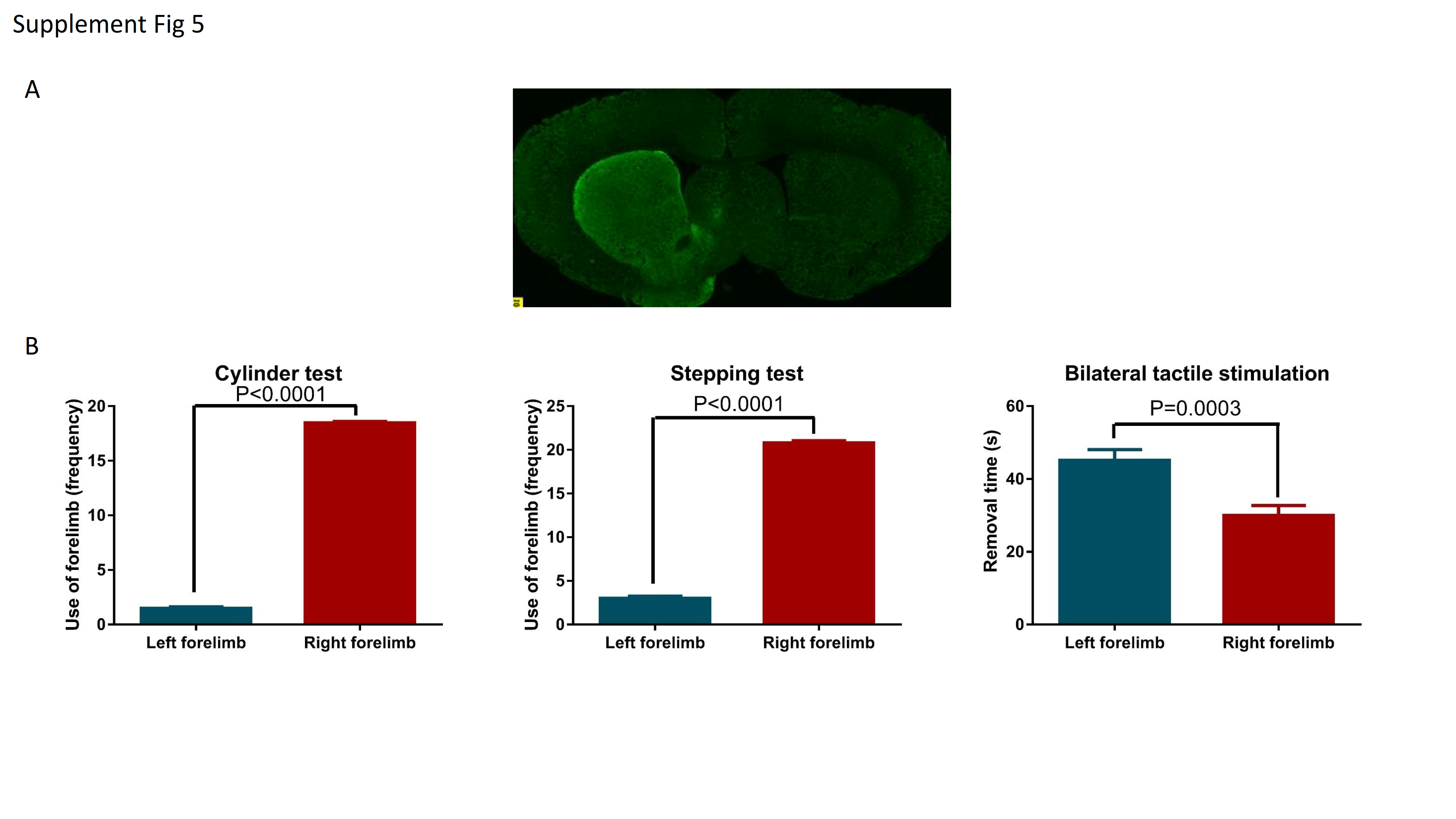

Supplement: Supplementary Figure 5 — The rat Parkinsonism model was induced by injection of 6-OHDA into the striatum, and damage to the striatum and substantia nigra (right side) was confirmed by (A) immunohistochemistry staining using an anti-TH antibody. The figure shows a representative immunohistochemistry staining in the 6-OHDA injected rat brain tissue. (B) The motor and sensing behavior of the model rats was examined by cylinder test, stepping test, and bilateral tactile stimulation assay. The data are collected from 58 6-OHDA injected rats (N = 58) and expressed as mean + SEM. [file Image_5.TIF]

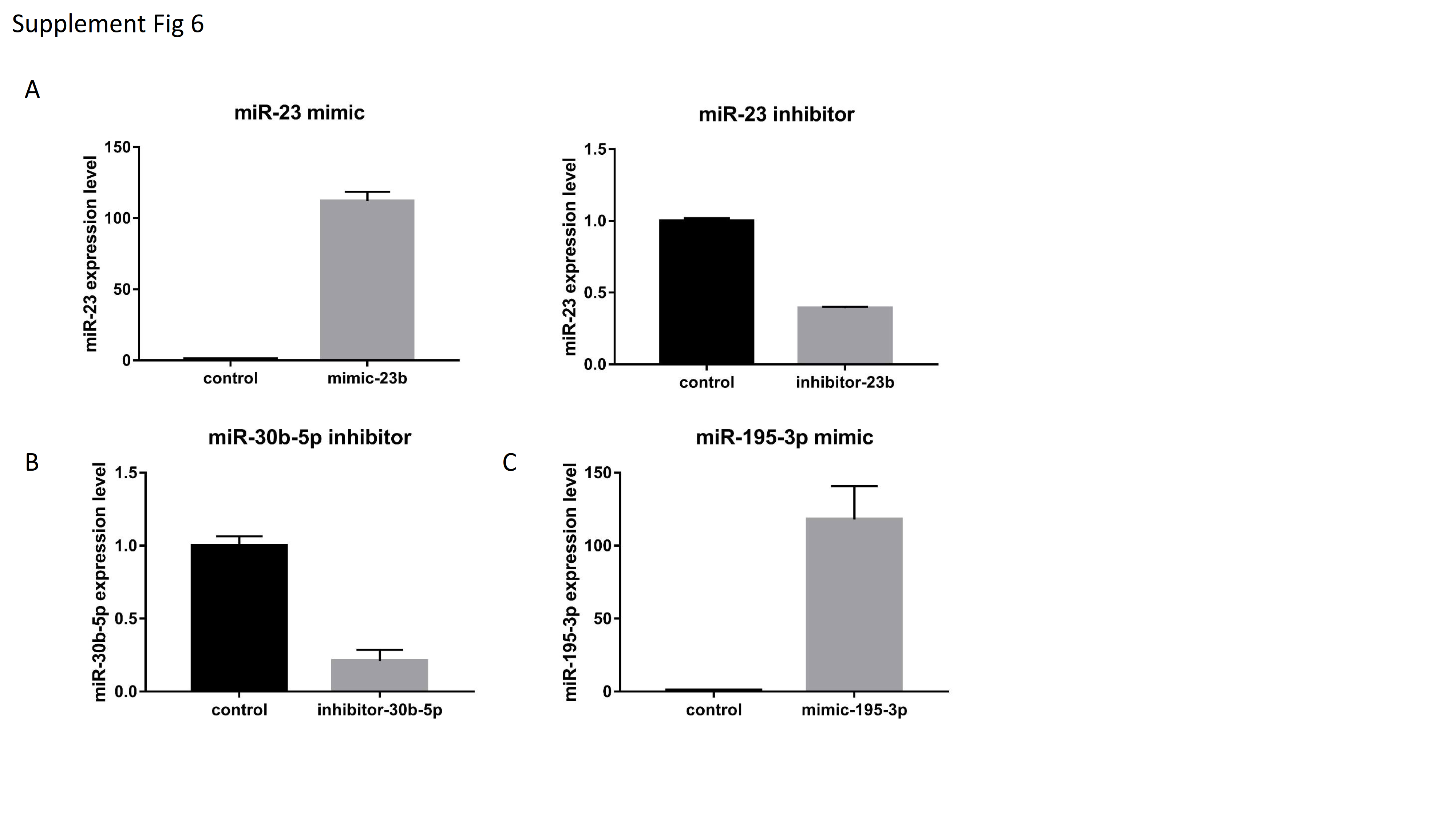

Supplement: Supplementary Figure 6 — NSCs were transfected with (A) miR-23b-3p mimic (mimic-23b-3p) or inhibitor (inhibitor-23b-3p), (B) miR-30b-5p inhibitor (inhibitor-30b-5p), and (C) miR-195-3p mimic (mimic-195-3p). The effects of inhibition or overexpression of miRNAs were measured by quantitative PCR. The data are collected from three independent experiments (N = 3) and expressed as mean + SEM. [file Image_6.TIF]
